# Proteomic changes in various organs of *Haemaphysalis longicornis* under long-term starvation

**DOI:** 10.1371/journal.pntd.0010692

**Published:** 2022-08-22

**Authors:** Ningmei Wang, Han Wang, Aimeng Ji, Ning Li, Guomin Chang, Jingze Liu, Desmond O. Agwunobi, Hui Wang

**Affiliations:** Ministry of Education Key Laboratory of Molecular and Cellular Biology, Hebei Key Laboratory of Animal Physiology, Biochemistry and Molecular Biology, College of Life Sciences, Hebei Normal University, Shijiazhuang, Hebei Province, China; Baylor College of Medicine, UNITED STATES

## Abstract

*Haemaphysalis longicornis* (Neumann), a tick of public health and veterinary importance, spend the major part of their life cycle off-host, especially the adult host-seeking period. Thus, they have to contend with prolonged starvation. Here, we investigated the underlying molecular mechanism of tick starvation endurance in the salivary glands, midguts, ovaries, and Malpighian tubules of starved *H*. *longicornis* ticks using the data-independent acquisition quantitative proteomic approach to study the proteome changes. Essential synthases such as glutamate synthase, citrate synthase, and ATP synthase were up-regulated probably due to increased proteolysis and amino acid catabolism during starvation. The up-regulation of succinate dehydrogenase, ATP synthase, cytochrome c oxidase, and ADP/ATP translocase closely fits with an increased oxidative phosphorylation function during starvation. The differential expression of superoxide dismutase, glutathione reductase, glutathione S-transferase, thioredoxin, and peroxiredoxin indicated fasting-induced oxidative stress. The up-regulation of heat shock proteins could imply the activation of a protective mechanism that checks excessive protein breakdown during starvation stress. The results of this study could provide useful information about the vulnerabilities of ticks that could aid in tick control efforts.

## Author summary

Ticks are a common blood-sucking parasite, which spread many pathogens that cause serious diseases such as Lyme disease to people. Ixodid ticks can take up to three blood meals in their life. During the long process of waiting for their host in the wild, they have evolved a strong ability to tolerate hunger, which should not take more than a year. To study these tenacious molecular regulatory mechanisms, we conducted the DIA quantitative proteomics technology to perform large-scale protein quantitative research on various tissues of *Haemaphysalis longicornis* starved for a long time. Through the analysis of thousands of proteins produced by the performed research, the results showed that many proteins in the ticks starved for a long time had expressed quantitative changes such as the increased expression of some synthase enzymes. The large amount of data provided by this study can help to better understand the molecular mechanism of ticks’ long-term hunger tolerance. Although this study focuses on finding possible mechanisms for tick starvation resistance at the protein level, the current findings may well have a bearing on research about special activities such as ultra long-distance space travel in the dormant state of the human body in the future.

## 1. Introduction

The Asian long-horned tick, *Haemaphysalis longicornis* Neumann, is a hard tick (Ixodidae) that is widely distributed across China, Korea, Japan, Australia, New Zealand, and some South Pacific Island Nations [1, 2]. In the United States of America, it was first identified in New Jersey in 2017 [3], and since then, it has been reported in 11 more states [4, 5]. *H*. *longicornis* acts as an important vector of diseases such as bovine theileriosis [6], babesiosis [7, 8], hemolytic anemia [9], spotted fever group rickettsiae (SFGR) [10, 11], and severe fever with thrombocytopenia syndrome virus (SFTSV) [12, 13]. Thus, *H*. *longicornis* is a vector of public health and veterinary importance.

One of the major stressors that ticks have to contend with during the off-host period is starvation, and this often presents the challenge of dehydration to hemaphatogous arthropods like ticks given that the blood meal contains a large amount of water [14]. Reduction in energy resources and significant challenges to ion and water homeostasis is associated with starvation stress [15]. As with other ixodid ticks, the life of *H*. *longicornis* comprises a multi-stage life cycle (larva, nymph, adult) with each stage depending on a blood meal for development and reproduction (for adult stage) [16]. Although ixodid ticks feed only three times in their life, their generation time can last for some years, which implies that their life cycle comprises of a starvation period [17]. Most ixodid ticks like *H*. *longicornis* can withstand prolonged starvation for an extended period of 1–2 years between feeding and it is the host-seeking period of the adult stage that seems to be the longest in their life cycle [17]. In other words, they have to spend the major part of their lives enduring starvation stress by relying on energy reserves acquired in the previous session of blood meal [18]. When the nutrient reserves are exhausted, they could employ autophagy and other physiological routes as survival strategies [17].

Although some ticks’ starvation-enduring strategies have been discussed in previous studies, however, information about the underlying molecular mechanisms of starvation endurance in ticks is scarce and remains unclear. In the present study, a data-independent acquisition (DIA) quantitative proteomic approach was employed to investigate the differential proteome changes in starved ticks in the hope of gaining further insight into how the tick *H*. *longicornis* withstands prolonged starvation.

## 2. Methods

### Ethics statement

Ethical guidelines of the institution were observed and all experimental protocols were approved by the Animal Ethics Committee of Hebei Normal University (Protocol Number: IACUC-157026).

## (a) Sample collection and tick rearing

The *H*. *longicornis* ticks used in this study were captured from the vegetation of Xiaowutai Mountain National Nature Reserve of Hebei Province, China. The ticks were allowed to feed on the ears of New Zealand white rabbits (*Oryctolagus cuniculus*) tied with earmuffs made of white cloth. Each rabbit was kept at constant room temperature (25 ± 1°C), and used for a single infestation. During the non-feeding period, the ticks were maintained in artificial climate incubators at 25 ± 1°C, 75% relative humidity, and 16/8 h of L/D cycle as described by J Liu, Z Liu, Y Zhang, X Yang and Z Gao [19]. Thereafter, hungry female ticks were selected for the starvation experiment.

### (b) Tick subjection to starvation, dissection, and protein extraction

Given that *H*. *longicornis* is relatively a small tick, to obtain sufficient proteins from the organs for this experiment, 80 unfed adult female ticks were used per group making it 320 ticks in total. Apart from the control group (newly molted adult), the experimental groups (A, B, and C) were subjected to starvation for 2, 4, and 6 months respectively. Thereafter, each tick was dissected in PBS containing protease inhibitor cocktail (Roche, Mannheim, Germany) at pH 7.2 and salivary glands, midguts, ovaries, and Malpighian tubules were collected, flash-frozen in liquid nitrogen, and stored at –80°C until protein extraction. The protein extraction protocol was performed as previously described [20]. The frozen samples were deposited in a pre-cooled glass homogenizer and ground in PBS buffer, and then the homogenate was transferred into a 15 ml centrifuge tube. The homogenate for each of the samples was centrifuged for 10 min (4°C, 12,000 × *g*). The supernatant was collected, transferred into a new 15 ml centrifuge tube, and 3 ml of Tris-saturated phenol (pH 7.8) was added to it. The mixture was vortexed for 1 min, centrifuged at 12,000 × *g* for 10 min at 4°C, and the aqueous phase was discarded. Then, 3 ml of 50 mM Tris-HCl with a pH of 8.0 was added and the mixture was thoroughly mixed (vortexed) for 1 min and centrifuged for 10 min (4°C, 12,000 × *g*). Thereafter, the upper aqueous phase was removed, and then for the protein content to be precipitated, 0.1 M ammonium acetate in methanol was added after which it was stored at –20°C overnight. Then, the protein was subjected to 10 min centrifugation (4°C, 12,000 × *g*) before the supernatant was discarded. The protein pellets were further washed with methanol, lyophilized, and stored at –80°C.

### (c) Protein digestion

The protein digestion was carried out as previously described [20]. The protein sample from each processing group was reduced in a 10 mM dithiothreitol solution at 37°C for 30 min, and alkylated with 20 mM iodoacetamide solution in the dark for 45 min at 25°C. Then, the proteins were digested at 37°C for 12 h with trypsin (1:50 w/w, ThermoFisher Scientific, USA). Thereafter, a C18 solid-phase extraction column (CNW, Anpel, China) was used to desalinate the digested peptides. Afterward, the normalization of the purified peptide concentration was performed to a common value using a BCA Protein Assay Kit (Pierce, Rockford, IL, USA). The digestion efficiency was monitored with LC-MS (Q Exactive HF, Thermo Fisher, Waltham, MA, USA).

### (d) DIA quantitative proteomic analysis

The salivary glands, midguts, ovaries, and Malpighian tubules of female ticks after different starvation (0 month, 2 month, 4 month, and 6 month) were quantitatively analyzed by DIA proteomics method. There were 4 sampling time points for each tissue, and the test was repeated 3 times, with a total of 12 samples. Therefore, there were 48 samples from the above three tissues. Each sample was resuspended in solvent A (99.9% H_2_O, 0.1% FA) containing iRT reagent (Biognosys Spectronaut, Switzerland). DIA quantitative analyses were performed using LC-MS with a UPLC M-Class system (Waters, USA) and a Q Exactive HF mass spectrometer (Thermo Fisher, Waltham, MA, USA). After loading each sample onto a C18 RP trap column (5 μm particle size, 100 Å pore size, 180 μm ID × 20 mm length; Waters, USA), they were separated on a C18 RP analytical column (1.8 μm particle size, 75 μm ID × 250 mm length; Waters, USA) at a flow rate of 300 nL/min with the linear ACN gradient as follows: 2–8% solvent B over 6 min and 8–35% solvent B over the next 114 min (solvent A: 99.9% H_2_O, 0.1% FA; solvent B: 99.9% ACN, 0.1% FA). The sample was electrosprayed into the Q Exactive HF mass spectrometer (voltage: 2.0 KV, heating capillary temperature: 290°C), and the parameters were set. DIA mode parameters of Q Extractive HF were set as follows: (a) scanning range was 350–1200 m/z; (b) resolution of the precursor ion was 60,000; (c) automatic gain control (AGC) target was 3×10^6^; (d) maximum ion injection time (maximum IT) was 50 ms; (e) 27% HCD normalized collision energy; (f) DIA method set as: Full MS (350 to 1250 m/z), followed by 20 DIA MSMS, DIA isolation window (IW) were 59.0 m/z, 25.0 m/z, 19.0 m/z, 17.0 m/z, 13.0 m/z, 13.0 m/z, 11.0 m/z, 12.0 m/z, 12.0 m/z, 11.0 m/z, 12.0 m/z, 9.0 m/z, 10.0 m/z, 10.0 m/z, 11.0 m/z, 10.0 m/z, 10.0 m/z, 9.0 m/z, 10.0 m/z, 10.0 m/z, respectively; then Full MS (350 to 1250 m/z), followed by 20 DIA MSMS (IW) were 10.0 m/z, 9.0 m/z, 10.0 m/z, 8.0 m/z, 9.0 m/z, 9.0 m/z, 10.0 m/z, 10.0 m/z, 10.0 m/z, 10.0 m/z, 9.0 m/z, 10.0 m/z, 10.0 m/z, 10.0 m/z, 10.0 m/z, 10.0 m/z, 11.0 m/z, 10 m/z, 10 m/z, 11 m/z, respectively; then Full MS (350 to 1250 m/z), followed by 20 DIA MSMS (IW) were 10 m/z, 11 m/z, 12 m/z, 11 m/z, 13 m/z, 13 m/z, 13 m/z, 14 m/z, 13 m/z, 14 m/z, 14 m/z, 19 m/z, 18 m/z, 20 m/z, 27 m/z, 24 m/z, 33 m/z, 45 m/z, 56 m/z, 91 m/z, respectively. (g) MSMS scan resolution was 30, 000, AGC target was 1×10^6^. Raw data were analyzed using version 15.0 Spectronaut software (Switzerland), and using the default parameters for DIA data analysis (FDR<1%). The database of the *H*. *longicornis* proteins derived from transcriptome sequencing (NCBI accession Number: GHLT00000000) was utilized for the DIA mass spectra searching. To exclude the possibility of result contamination, the rabbit (*Oryctolagus cuniculus*) and human keratin sequences were used as the contaminated database for proteomic searching. The selected search parameters were as follows: (i) trypsin digestion with 2 missed sites; (ii) N-terminal acetylation, variable modifications set methionine oxidation; (iii) fixed modifications set carbamidomethylation of cysteine. The proteomics data from the mass spectrometry was deposited to the ProteomeXchange Consortium (http://proteomecentral.proteomexchange.org) through the iProX partner repository with the dataset identifier PXD031596. Proteins between different groups containing at least two unique peptides, a Q value <0.05, and an expression change value >2-fold were considered to have significant expression changes (<0.5 is down-regulated and >2 is up-regulated), which was the basis for further data analysis and discussion.

### (e) Bioinformatic analysis

All the differentially expressed proteins were subjected to bioinformatics analysis. GProX was used for clustering the proteins with similar expression change patterns [21]. The number of clusters was set to 5, and the regulation threshold was fixed at 1 and –1 for protein up-regulation and down-regulation, respectively, which corresponds with the original ratios of 2 and 0.5, respectively. The principal component analysis (PCA), volcano plots intersection graph, and KEGG figures were drawn using the online analysis software Omicsolution (https://www.omicsolution.org/wkomics/main/). At the same time, omicsolution was also used to obtain the Gene Ontology (GO) functional, while the pathways associated with the differential protein expressions were identified with the Kyoto Encyclopedia of Genes and Genomes (KEGG) database (http://www.kegg.jp/kegg/).

## 3. Results

### (a) Protein identification summary

**Fig 1:**
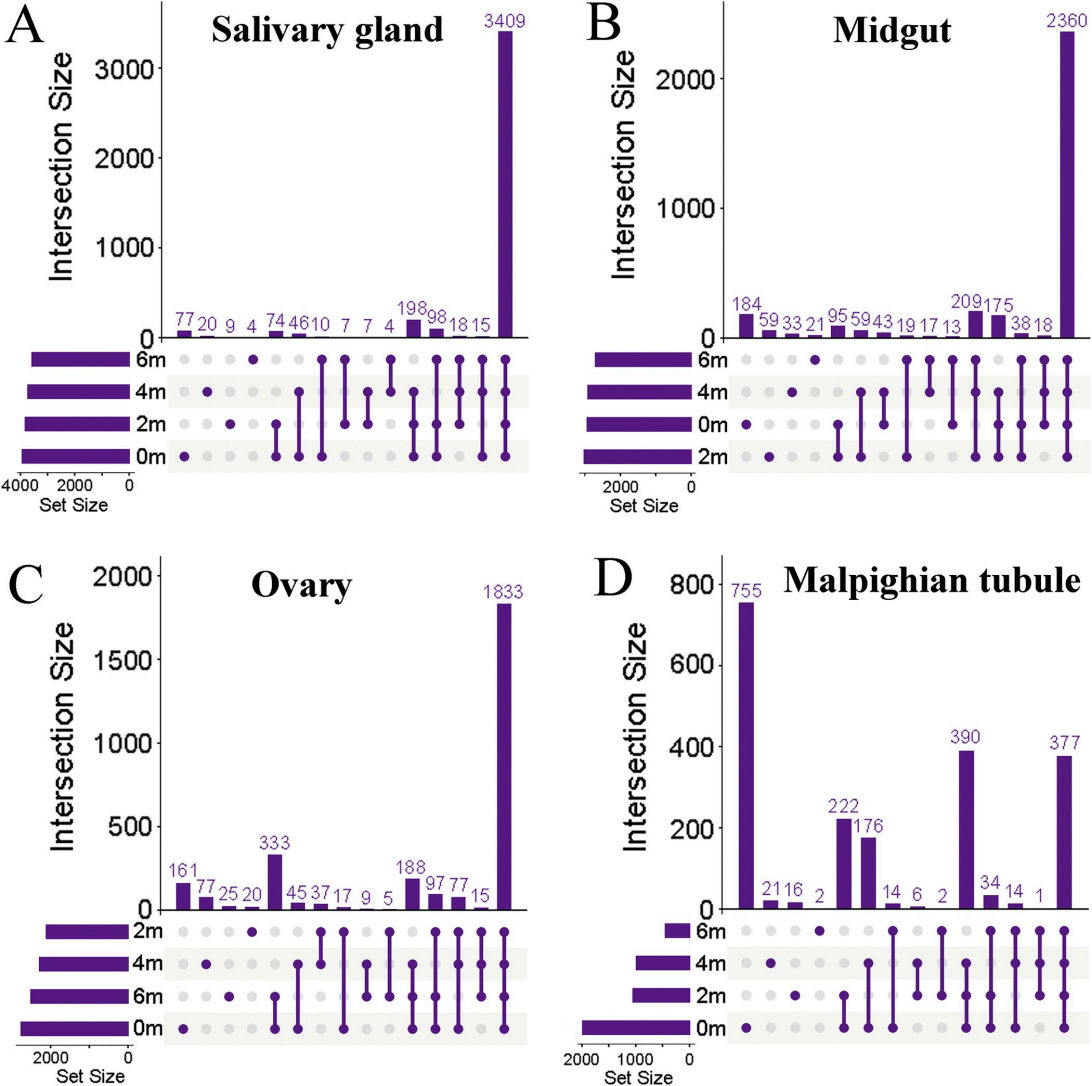
Analysis of four tissue intersection proteins at different stages of starvation of female *H*. *longicornis*. The dot represents the individual proteins in the four periods, the dot and the connecting line represent the intersection proteins in different periods, and the bar graph corresponding to it on the top represents the number of proteins. A. Salivary glands, B. Midguts, C. Ovaries, D. Malpighian tubules. m: month.

S1 Fig shows the experimental workflow of the DIA quantitative proteomics. After the ticks were subjected to prolong starvation periods, the proteins were extracted, digested with trypsin, subjected to LC/MS identification and data analysis, the proteins were identified and quantitative results were obtained. A total of 4016 proteins (S1 Table) were identified in the salivary glands, 3513 proteins (S2 Table) were identified in the midguts, 3074 proteins (S3 Table) were identified in the ovaries, and 2382 (S4 Table) proteins were identified in the Malpighian tubules. Furthermore, the proteins that can be identified in each repeat were counted, and other proteins with filtered values were filtered out. The intersection graph showed the overlap of proteins from four groups (experimental and control groups) (Fig 1A–1D). A total of 3409, 2360, 1833, and 377 high-confidence proteins were shared among all the four groups in the salivary glands (Fig 1A), midguts (Fig 1B), ovaries (Fig 1C), and Malpighian tubules (Fig 1D), respectively.

### (b) PCA and proteome analysis

**Fig 2:**
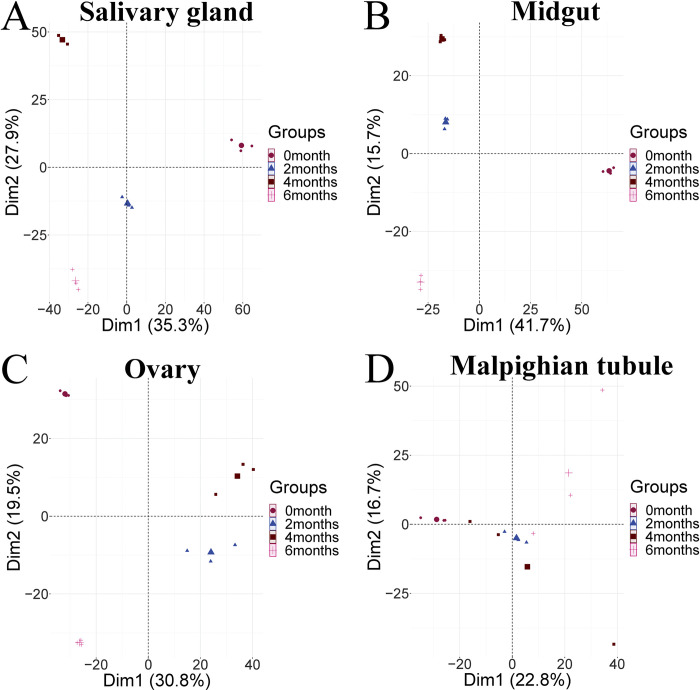
PCA analysis of four tissues in different periods of starvation of female *H*. *longicornis*. A. Salivary glands, B. Midguts, C. Ovaries, D. Malpighian tubules.

**Fig 3:**
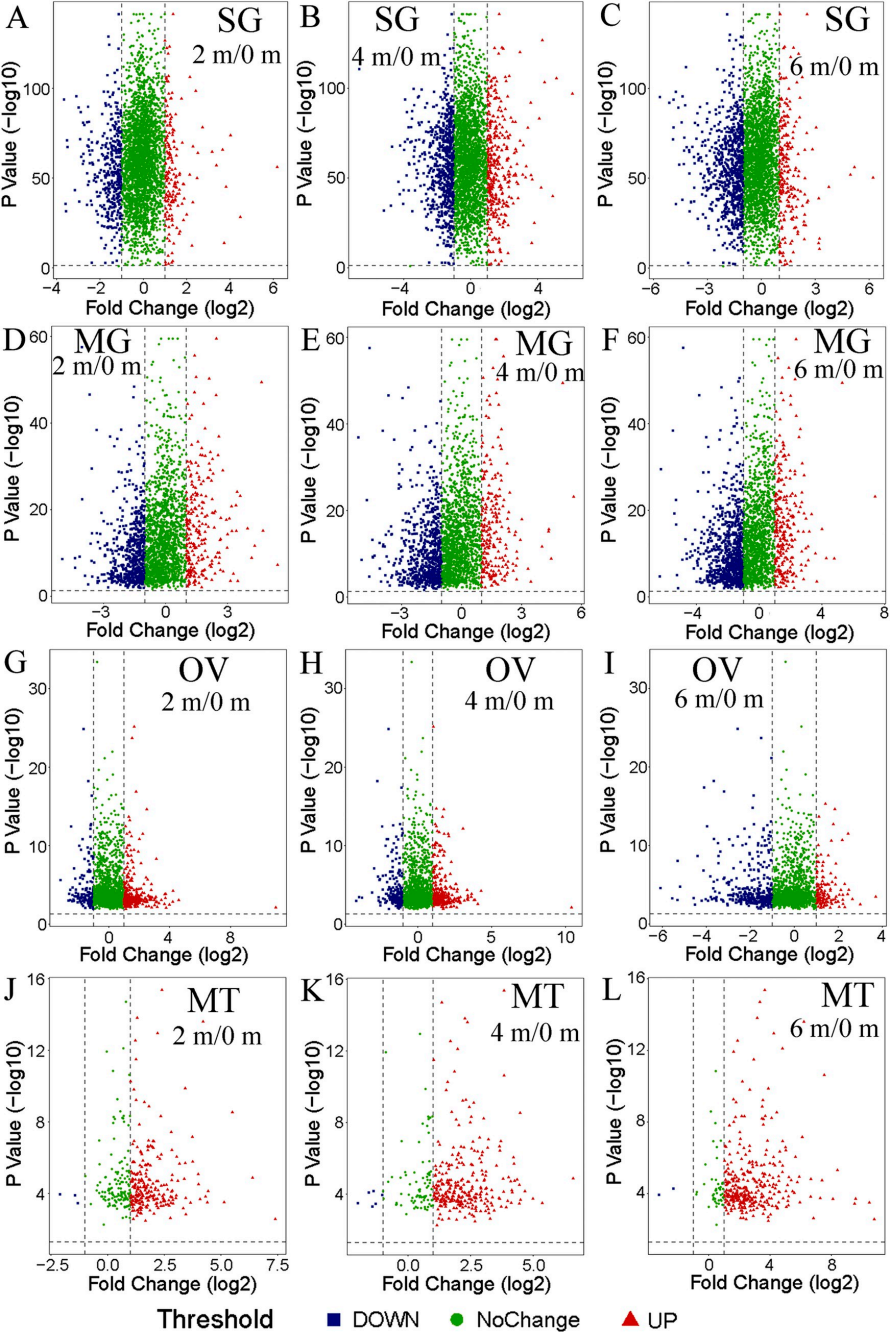
Volcano plot analysis of four tissues of female *H*. *longicornis* in different periods of starvation. The red triangle represents up-regulation whereas the blue square represents down-regulation, and the green circle represents no change. SG: Salivary glands; MG: Midguts; OV: Ovaries; MT: Malpighian tubules. 0 m: 0 month (control), 2 m: 2 months, 4 m: 4 months, 6 m: 6 months. A-C. Salivary glands, A: 2 m/0 m, B: 4 m/0 m, C: 6 m/0 m; D-F. Midguts, D: 2 m/0 m, E: 4 m/0 m, F: 6 m/0 m; G-I. Ovaries, G: 2 m/0 m, H: 4 m/0 m, I: 6 m/0 m; J-L. Malpighian tubules. J: 2 m/0 m, K: 4 m/0 m, L: 6 m/0 m.

To evaluate the quality of the proteome data, principal component analysis (PCA) was performed using the four replicates of each treatment group (Fig 2), which indicated high reproducibility of the repeated experiments of each group. Take the salivary glands (Fig 2A) as an example, the result showed significant differences between the experimental groups and the control group, whereas the differences among the four replicates within each group were smaller. To display changes in the expression levels of all the identified proteins at different levels/months of starvation compared with those at 0 months, the volcano plots (Fig 3) were constructed, the plots showed that there were more differentially expressed proteins in the salivary glands (Fig 3A) and midguts (Fig 3B). With the prolongation of starvation time, the number of down-regulated proteins in ovaries was increasing (Fig 3C), however, there were more up-regulated proteins in the Malpighian tubules (Fig 3D).

### (c) Cluster analysis of differentially expressed proteins

**Fig 4:**
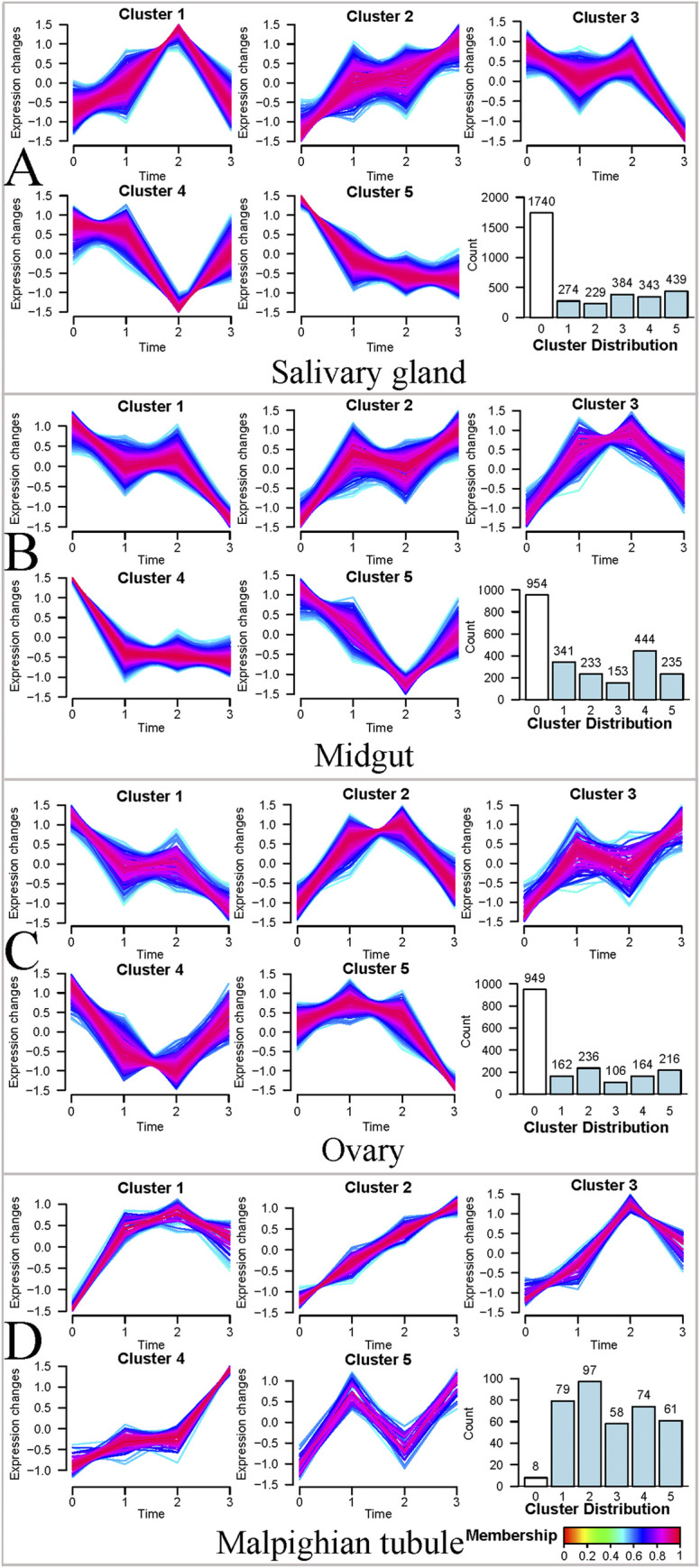
Cluster analysis of intersected proteins in four tissues of female *H*. *longicornis* at different stages of starvation. The intersected proteins were divided into 5 categories, and 0 represents the number of proteins with no change. A. Salivary glands, B. Midguts, C. Ovaries, D. Malpighian tubules.

Cluster analysis was performed using proteins that intersected all groups in the salivary glands (3409 proteins), midguts (2360 proteins), ovaries (1833), and Malpighian tubules (377 proteins) (Fig 4). Through GProX Platform, proteins in each tissue were divided into 5 expression types: continuous up-regulation, continuous down-regulation, down-regulation after up-regulation, up-regulation after down-regulation, and irregular change. In this way, a large number of protein expression trends can be clearly classified during the long-term starvation tolerance process of ticks, and the understanding of protein expression characteristics is strengthened. For the salivary glands (Fig 4A), 1740 proteins exhibited no significant changes in expression, whereas 1669 proteins showed significant changes in their expression levels. For the midguts (Fig 4B), 954 proteins showed no significant changes in expression levels, while 1406 proteins had significant changes. In the ovaries (Fig 4C), 949 proteins showed no significant changes in expression levels, but 884 proteins were significant changes. From the 377 proteins in the Malpighian tubules (Fig 4D), only 8 proteins showed no significant changes in protein expression levels, whereas 369 proteins showed significant changes in their expression levels. There were variations in the change trends for the differentially expressed proteins (DEPs). In salivary glands (Fig 4A), 229 proteins showed a continuous up-regulation trend in cluster 2; 439 proteins continued to decrease in cluster 5, while cluster 3 showed that 384 proteins remained stable from 0 to 4 months of starvation, and began to decrease sharply from 4 to 6 months. In the midguts (Fig 4B), although 341 proteins fluctuated between 2 and 4 months of starvation, they generally showed a downward trend, while 233 proteins generally showed an upward trend, and 444 proteins decreased significantly after 2 months of starvation, This indicates that *H*. *longicornis* may alleviate the adverse effects of hunger through the continuous degradation of these proteins, so that it can survive better. In the ovaries (Fig 4C), the proteins of cluster 1 and cluster 3 showed similar trends as those of cluster 1 and cluster 2 in the midguts. 164 proteins in cluster 4 remain stable in the early stage of starvation, and decline sharply from the 4th month to the 6th month of starvation, indicating that these proteins may have important functions *in vivo* and remain stable in the early stage. In the Malpighian tubules (Fig 4D), 97 proteins showed a significant upward trend, and 79 proteins began to up-regulate rapidly in the early stage of hunger, and maintained a high level, indicating that these proteins may have important value in resisting hunger. Although all proteins in the Malpighian duct fluctuate, they generally showed an upward trend, which may be related to its excretory function.

### (d) Function annotation (GO) of the DEPs

**Fig 5:**
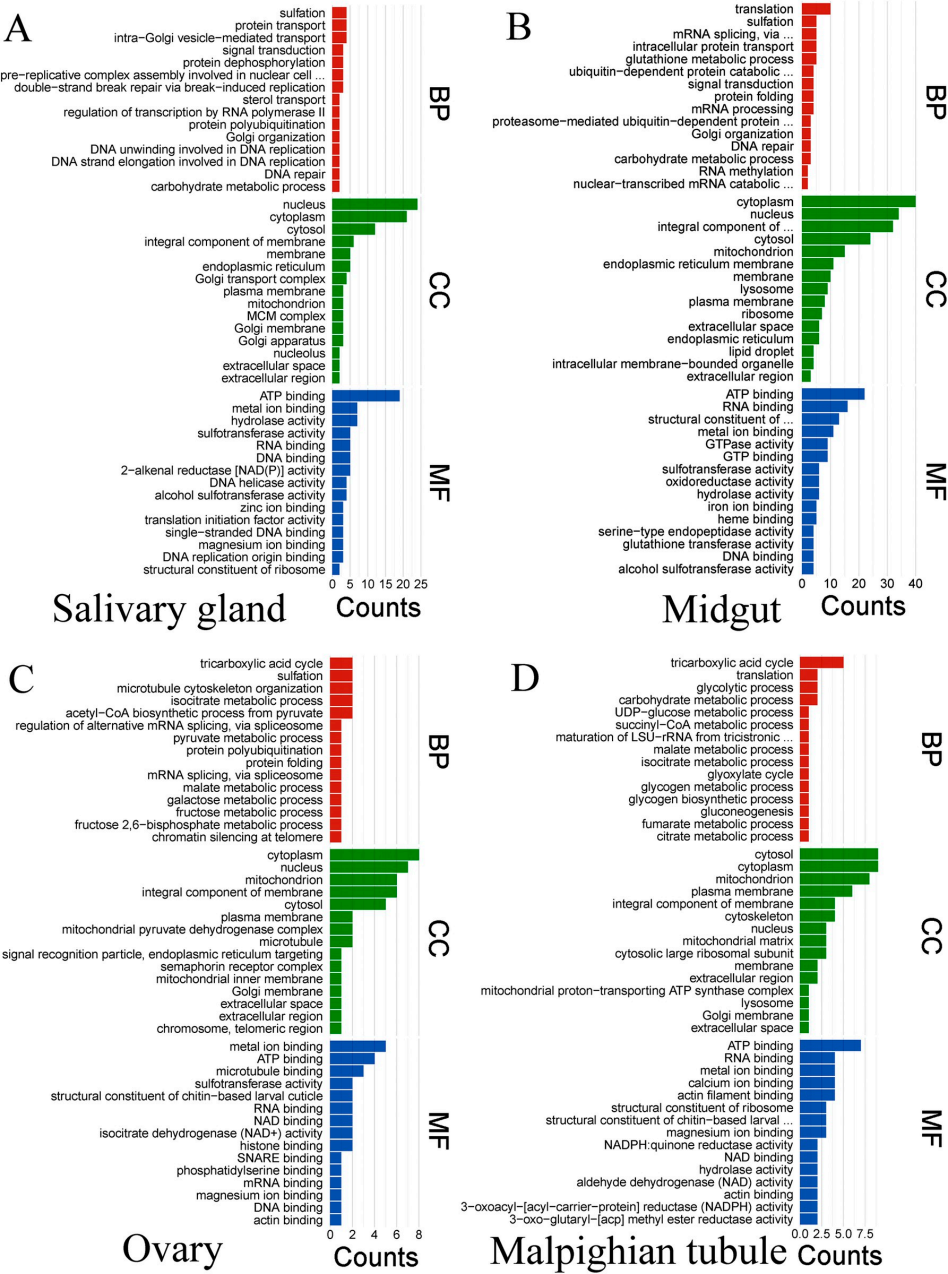
Gene ontology (GO) enrichment analysis of differentially expressed proteins in four tissues of female *H*. *longicornis* at different stages of starvation. BP: Biological process; CC: Cellular component; MF: Molecular function. **A.** Salivary glands, B. Midguts, C. Ovaries, D. Malpighian tubules.

Gene ontology (GO) enrichment analysis was performed to show the functions of the DEPs responding to starvation stress. The DEPs-associated functional categories in response to starvation stress are shown in Fig 5. In salivary glands (Fig 5A), 146 terms were enriched in biological processes, of which sulfation, protein transport, and intra Golgi vesicle mediated transport were the most enriched. In the molecular function category, 153 terms were enriched, of which 19 proteins were enriched in ATP binding. 74 terms were enriched in the cellular component, and 24 proteins were in the nucleus term (Fig 5A; S5 Table). 237 terms of biological processes were enriched in the DEPs of the midguts (Fig 5B), of which 10 proteins are involved in the process of translation. A total of 252 terms were enriched in molecular functions, most of which were ATP binding, RNA binding, structural constitution of ribosome, and metal binding. 103 terms were enriched in the cellular component, of which 40 of these proteins were enriched in cytoplasm (Fig 5B; S6 Table). In the ovaries (Fig 5C), 58 terms were enriched in biological processes and 61 terms were enriched in molecular functions, of which metal ion binding accounted for more proteins, and 34 terms were enriched in the cellular component. Cytoplasm term accounted for a large proportion DEPs in the cellular component (Fig 5C; S7 Table). Tricarboxylic acid cycle accounts for the largest proportion of DEPs in the biological processes of Malpighian tubules (Fig 5D). A total of 130 terms were enriched in the molecular function of Malpighian tubules, and 35 terms were enriched in the cellular component, among which the most DEPs were in cytosol and cytoplasm (Fig 5D; S8 Table).

### (e) KEGG pathway analysis of DEPs

**Fig 6:**
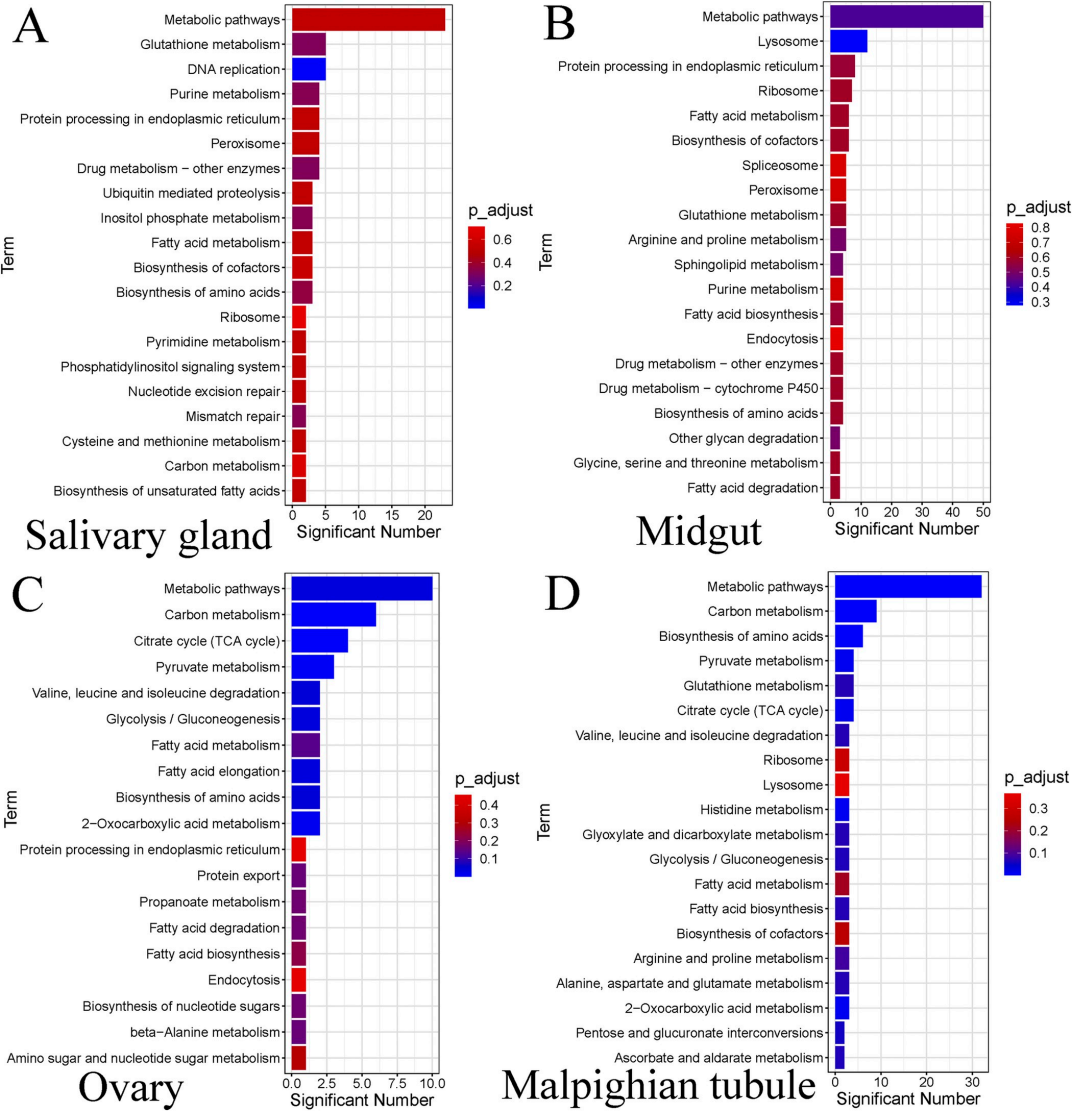
KEGG pathway enrichment analysis of differentially expressed proteins in four tissues of female *H*. *longicornis* at different stages of starvation. Significant Number represents the number of proteins in each pathway. A. Salivary glands, B. Midguts, C. Ovaries, D. Malpighian tubules.

The differentially expressed proteins were subjected to KEGG pathway enrichment analysis (Fig 6). The most significantly enriched KEGG pathways in the salivary glands (Fig 6A; S9 Table) include “Metabolic pathways”, “DNA replication”, “Glutathione metabolism”, “Drug metabolism-other enzymes”, and “Purine metabolism”, among others. In the midguts (Fig 6B; S10 Table), the most enriched pathways include “Metabolic pathways”, “Lysosome”, “Protein processing in endoplasmic reticulum”, “Ribosome”, “Fatty acid metabolism”, and “Biosynthesis of cofactors”, among others. The most significant KEGG pathways enriched for DEPs in the ovaries (Fig 6C; S11 Table) pertained to “Metabolic pathways”, “Carbon metabolism”, “Citrate cycle (TCA cycle)”, “Pyruvate metabolism”, and “2-Oxocarboxylic acid metabolism”, among others. In the Malpighian tubules (Fig 6D; S12 Table), the “Metabolic pathway” was the most enriched pathway. Other significantly enriched pathways include “Carbon metabolism”, “Biosynthesis of amino acids”, and “Citrate cycle (TCA cycle)”. “Metabolic pathway”, which is the most abundant pathway in all the organs indicates that many metabolism-related proteins play an important role in the starvation tolerance of *H*. *longicornis*.

## 4. Discussion

Ticks spend the major part of their lives off-host where they have to rely on energy reserves obtained from the previous blood-feeding session, and the challenge of starvation arises once the energy reserve is depleted [18]. Unlike most arthropods, ticks are obligate blood-feeding organisms, have to endure a period of about 1 to 2 years between feedings [22]. To survive for a long period, they have to contend with diverse environmental stressors such as starvation and dehydration. Here, we investigated the proteome changes in *H*. *longicornis* ticks in response to prolonged starvation to gain further insight into how ticks survive prolonged starvation during the long-term off-host period.

Vital synthases such as glutamate synthase, citrate synthase, ATP synthase, and ATP-citrate synthase were up-regulated in the Malpighian tubules. Also, glutamate synthase was up-regulated in the ovaries and salivary glands. ATP synthase was up-regulated in the salivary glands (Table 1). Notably, increased proteolytic activities and amino acid catabolism take place during starvation to enhance the production and addition of glutamine into circulation [23]. Glutamine is essential for the reactivation of mTORC1 by autophagy during amino acid starvation [24]. The up-regulation of glutamate synthase could be associated with glutamine-glutamate metabolism. The conversion of glutamine to glutamate and restoration of non-essential amino acid (NEAA) pool culminate in mTORC1 reactivation [24]. Unlike in the Malpighian tubules and ovaries, there was no significant changes in citrate synthase (a key mitochondrial enzyme) expression in the salivary glands and midguts. Similarly, there were no significant changes in the expression of citrate synthase in some tissues in the invertebrate *Sepia officinalis*, such as gill tissue [25]. However, *S*. *officinalis* was fasting for only 12 days. If *S*. *officinalis*, like the ticks in this experiment, is starved for several months, a similar phenomenon may occur. It is not clear why the expression patterns of citrate synthase in the salivary glands were different from that of other tissues. Further investigation is required to get a possible explanation for this differential expression pattern. Most of the proteins synthesized by these synthases as aforementioned were involved in energy production. Although the expression of these proteins found in this study has not been clearly reported in the other research about long-term starving ticks, the mRNA expression of some genes (such as 6-phosphofructo-2-kinase/fructose 2,6-bisphosphatase) related to carbohydrate metabolism in the long-term starved tick *Dermacentor marginatus* was significantly increased. Most of these proteins were also involved in glycolysis and other cycle reactions related to energy production, but unfortunately, the tissue-specific expression was not clearly given, so it is impossible to accurately compare them with the significantly up-regulated proteins found in this study [26].

**Table 1:** Selected differentially expressed proteins during starvation in *H*. *longicornis*.

Protein names	ID	organs	Expression levels of proteins		
			2 m/0 m	4 m/0 m	6 m/0 m
Synthases					
Glutamate synthase	m.32109	SG	–	–	↑
		OV	↑	–	↑
		MT	–	↑	↑
Citrate synthase	m.24599	OV	↑	↑	–
		MT	↑	↑	↑
ATP synthase	m.11600	SG	↑	↑	–
		OV	↑	↑	↓
	m.11980	MT	↑	↑	↑
ATP-citrate synthase	m.39732	MT	–	↑	↑
Mitochondrial oxidative phosphorylation					
Succinate dehydrogenase	m.32953	OV	↑	↑	–
		MT	–	↑	↑
Cytochrome c oxidase	m.11993	SG	↑	↑	–
	m.3932	OV	↑	↑	↓
ADP/ATP translocase	m.20555	SG	↑	↑	–
		OV	↑	↑	–
		MT	↑	↑	↑
Antioxidant enzymes					
Superoxide dismutase	m.45483	MT	↑	↑	↑
Glutathione S-transferase	m.15854	MT	↑	↑	↑
Thioredoxin	m.22450	MG	↑	↑	↑
		MT	↑	–	↑
	m.40240	MT	↑	↑	↑
Thioredoxin reductase	m.33183	MG	↑	↑	↑
		MT	↑	↑	–
	m.34237	OV	↑	↑	↑
Peroxiredoxin	m.24185	MT	↑	↑	↑
Other important proteins					
Ferritin	m.8017	SG	↑	↑	↑
		MG	↑	↑	↑
		OV	↑	↑	–
		MT	–	↑	↑
Vitellogenin-1	m.43085	SG	–	–	↓
		MG	–	–	↓
		OV	↓	↓	↓
		MT	↑	↑	↑
Vitellogenin-2	m.22646	SG	↓	↓	↓
	m.6201	MG	–	↑	↓
	m.39719	OV	↓	↓	–
		MT	–	–	↑
Heat shock protein	m.15080	SG	–	↑	↑
	m.10577	MG	↑	↑	↑
	m.15080	OV	↑	↑	↑
	m.10577 m.14185 m.10939 m.38491 m.34084 m.26040	MT	↑	↑	↑

SG: Salivary glands; MG: Midguts; OV: Ovaries; MT: Malpighian tubules; ↑: up-regulated; ↓: down-regulated;–: no significant change.

ATP synthase and succinate dehydrogenase are crucial mitochondrial oxidative phosphorylation enzymes. Both were up-regulated in the Malpighian tubules and ovaries (6 m down-regulated) of starved *H*. *longicornis* ticks in the present study. This was corroborated in a study where succinate dehydrogenase, ATP synthase, and cytochrome c oxidase genes were up-regulated after three weeks of starvation in *Paralichthys adspersus* [27]. However, there was no significant increase in ATP synthase expression in the fat bodies of the blood sucking insect *Panstrongylus megistus*, which had been starved for 7 days [28]. This may be related to tissue specificity. ATP-dependent processes in the liver (gluconeogenesis, β-oxidation of fatty acids, and ureagenesis) which supply ketogenic substrates are activated by starvation [29]. This stimulation of oxidative phosphorylation by long-term starvation could be a function of metabolic adaptation involving elevated rate of lipolysis, ketogenesis, increased hepatic fatty acid oxidation, and reduced uptake of glucose and oxidation in peripheral tissues as observed in long-term starved *Sparus aurata* [30]. A similar phenomenon also occurred in the long-term starved tick *D*. *marginatus*. In the study of the long-term starved tick *D*. *marginatus*, the transcription levels of mRNAs of proteins associated with metabolism and lipid metabolism plus the mRNAs of carbohydrate metabolism related proteins, were also up-regulated [26]. Hence, this elevated oxidative phosphorylation activity during prolonged starvation enhances substrate oxidation with the corresponding ATP supply and this could be responsible for the ability of most ticks to survive more than a year without blood acquisition. Further studies are required for an in-depth understanding of the dynamics of ATP synthase and succinate dehydrogenase expression during starvation.

Other important proteins involved in the mitochondrial oxidative phosphorylation that were showing an up-regulated trend in *H*. *longicornis* salivary glands and ovaries include Cytochrome c oxidase (COX) and ADP/ATP translocase (ADPT) (Although the expression of ADP/ATP translocase was labile. ADPT was also up-regulated in the Malpighian tubules.). This was consistent with the up-regulation of COX and ADPT in the liver of *S*. *aurata* after prolonged starvation [30]. The up-regulation of these proteins closely fits with an increased oxidative phosphorylation function during starvation. The up-regulation of COX could be based on the hypothesis of the relevance of COX6A2 expression during starvation which was supported by animal sensitivity to food deprivation and the accompanying loss of weight [31]. The expression of ADP/ATP translocase was unstable in this study as it was up-regulated on the second and fourth month but the expression of this protein at the sixth month of starvation was restored to the same level as that in 0 month. A possible explanation could be based on the fact that the expression of ADPT is in proportion to the level of respiratory activity of the tissue [32]. In addition, availability of different ADPT proteins may not necessarily display functional differences but rather play a key role in regulating their expression levels depending on the energy requirement and variety of stimuli [33].

In the Malpighian tubules of *H*. *longicornis* tick, all the heat shock proteins (HSPs) detected in the present study were highly up-regulated throughout the starvation period, indicating the vital role of the HSPs during starvation stress. We found that the high expression of HSPs found in this study were consistent with the significant increase of transcription levels of almost all mRNAs of HSPs (60, 70, 90, small heat shock protein) found in long-term starving tick *D*. *marginatus* [26]. This strongly corroborates the high expression of HSPs in previous studies during prolonged food deprivation in insects [34–36]. It was reported that *Rhodnius prolixus*, a hematophagous insect, died prematurely within 32–40 days after the knockdown of HSP70 and subjection to starvation, compared to the control group [35]. The mortality of the insects may be due to the inability to resist starvation stress caused by the absence of the HSP or the inability to tolerate the increased hydric stress due to the lack of water associated with the long period of blood meal deprivation [37]. HSPs serve as molecular chaperones that maintain the structure of essential proteins and preserve enzymatic functions, especially during different stress conditions [38]. During prolonged starvation stress, there is breakdown/damage of vital proteins, and HSPs serve as key sensors, which leads to an increase in more HSP production. These HSPs fix misfolded key proteins and restore their conformation; thus, the increase in the expression of HSPs acts as a protective mechanism that checks excessive protein breakdown [39]. Unlike most insects, ticks have an extra-ordinary ability to withstand long period of starvation and a possible explanation could be the high expression profile of their numerous HSPs during prolonged starvation stress as reflected in the present study.

A growing body of scientific reports has indicated that prolonged fasting-induced oxidative stress in higher organisms is often marked by the up-regulation of most antioxidant enzymes [40–44]. In this study, the antioxidant enzymes were differentially expressed in *H*. *longicornis* ticks subjected to prolonged starvation and they include superoxide dismutase (SOD), glutathione reductase (GR), glutathione S-transferase (GST), thioredoxin, thioredoxin reductase, and peroxiredoxins (Prx). Unlike in the salivary glands, SOD was up-regulated throughout the fasting period in the Malpighian tubules, an expected result given the excretory and homeostatic role of the Malpighian tubules. This result is consistent with the increased activity of SOD in the liver of fish deprived of food for 5 weeks [40]. To further corroborate these findings, a study performed with the tick *D*. *marginatus* subjected to long-term starvation exhibited antioxidant response via the up-regulation of mRNA of antioxidant genes including SOD, GST, catalase, and glutathione peroxidase [26]. When starvation prolongs and exceeds the tolerance threshold of cells, the resultant accumulation of reactive oxygen species (ROS) in the mitochondria leads to extensive autophagy. To inhibit this large-scale autophagy, the cells activate a rapid response antioxidant defense mechanism mediated by endogenous antioxidant enzymes such as SOD, and GST, among others [43]. The increase of SOD can prolong the lifespan of SOD-deficient *Drosophila melanogaster* and improve their tolerance to oxidative stress [45]. At the same time, the study of invertebrate *Caenorhabditis elegans* showed that the life span of oxidative stressed nematodes could be prolonged by providing antioxidants [46]. Unsurprisingly, a study that investigated the physiological and transcriptomic shifts caused by prolonged starvation of *D*. *variabilis* ticks reported high proteolytic and autophagic activities which facilitate the mobilization of endogenous nutrients [47]. Additionally, Prx was up-regulated in the Malpighian tubules throughout the length of the starvation period (2, 4, and 6 months). Although the role of Prx has been overshadowed by other popular oxidative stress defense enzymes such as SOD, it is a widely distributed peroxidase and arguably the most essential peroxide and peroxynitrite scavenging enzyme in biology [48, 49]. A study unraveled another vital function of Prxs as facilitators of insulin biosynthesis and glucose-induced insulin secretion in insulin-secreting INS-1E cells [50]. When peroxiredoxin 4 (Prdx4) is overexpressed in glucose-responsive insulin-secreting INS-1E cells, it significantly utilized luminal hydrogen peroxide (H_2_O_2_) to improve the glucose-induced insulin secretion with the corresponding facilitation of proinsulin mRNA transcription [50]. This role of Prxs may significantly contribute to the sustenance of the ticks’ energy level to carry out the basic metabolic activity during the prolonged starvation period.

Ferritin is an iron storage protein that is vital to the blood-feeding and reproduction success in *H*. *longicornis* [51]. It was up-regulated in most of the organs in the present study. A possible explanation could be that the partial degradation of ferritin to release iron during stress with the corresponding increase in the iron levels, influences the activity of iron-responsive proteins which in turn increase translation of ferritin [52, 53]. The increased expression of ferritin mRNA in long-term starving tick *D*. *marginatus* has been found [26]. Our results further confirmed this conclusion from the protein level. The main function of FTL (ferritin light chain) is iron storage and its synthesis is regulated on both transcriptional and translational levels [53]. Additionally, the up-regulation of ferritin may have been induced by the oxidative stress generated by prolonged starvation given that a previous study demonstrated the essential antioxidant role of ferritins in *H*. *longicornis* tick [54].

Ticks have strong reproductive capacity. A female tick can produce thousands of eggs. In the present study, vitellogenin-1 (Vg-1) and vitellogenin-2 (Vg-2) (two major hemolymph proteins of *H*. *longicornis* ticks, which are also the main precursors of egg yolk in ticks via the process known as vitellogenesis) were down-regulated in the ovaries and midguts of starved *H*. *longicornis* tick. Blood feeding induces vitellogenesis in ticks, and Vg synthesis is deemed to take place in the fat body [55]. At the same time, the down-regulation of vitellogenin is consistent with a previous study where starvation significantly reduced the hemolymph levels of vitellogenin in starved Lubber grasshopper [55]. There is a strong correlation between adult nutrition and reproductive output in many arthropods [56, 57]. This has been demonstrated in hematophagous insects such as mosquitoes [58], and triatomid bugs [59], where egg production depended on adult feeding. Similarly, ticks require a blood meal for reproduction and the synthesis of vitellogenin depends on the nutrients derived from the blood [60].

## Conclusion

Ticks can survive a long period of starvation, unlike most arthropods. This study gave insight into the implications of the changes in the proteomes of *H*. *longicornis* tick in response to starvation using a quantitative proteomic approach. Although there were some other differentially expressed proteins whose role during starvation was unknown, this study investigated the starvation-induced protein expression and the roles of some essential proteins classes including mitochondrial oxidative phosphorylation proteins, storage proteins, molecular chaperones/housekeeping proteins, antioxidation enzymes, and a reproductive protein. The results of this study help in the understanding of the molecular adaptation of ticks during prolonged starvation and in addition could provide insights into tick vulnerabilities that may aid in tick control efforts.

### Data accessibility statement

All mass spectrometry proteomics data have been deposited to the ProteomeXchange Consortium (http://proteomecentral.proteomexchange.org) via the iProX partner repository with the dataset identifier PXD031596.

## Supporting information

S1 Fig The overall experimental workflow for the quantitative proteomic analysis of total protein changes in key organs of starved *H*. *longicornis*. (TIF)

S1 Table The original search results of salivary glands, the identification number of four periods, intersection protein, and the differential multiple of protein expression. 0 m: 0 month (control), 2 m: 2 months, 4 m: 4 months, 6 m: 6 months. The p-value that was the basis for the q-value calculation. The p-value is calculated was the inverse cumulative distribution function of the decoy distribution for a given C-score. Calculated by T test.
(XLSX)

S2 Table The original search results of midguts, the identification number of four periods, intersection protein, and the differential multiple of protein expression. 0 m: 0 month (control), 2 m: 2 months, 4 m: 4 months, 6 m: 6 months. The p-value that was the basis for the q-value calculation. The p-value is calculated was the inverse cumulative distribution function of the decoy distribution for a given C-score. Calculated by T test.
(XLSX)

S3 Table The original search results of ovaries, the identification number of four periods, intersection protein and the differential multiple of protein expression. 0 m: 0 month (control), 2 m: 2 months, 4 m: 4 months, 6 m: 6 months. The p-value that was the basis for the q-value calculation. The p-value is calculated was the inverse cumulative distribution function of the decoy distribution for a given C-score.
(XLSX)

S4 Table The original search results of Malpighian tubules, the identification number in four periods, the intersection protein and the differential multiple of protein expression. 0 m: 0 month (control), 2 m: 2 months, 4 m: 4 months, 6 m: 6 months. The p-value that was the basis for the q-value calculation. The p-value was calculated as the inverse cumulative distribution function of the decoy distribution for a given C-score. Calculated by T test.
(XLSX)

S5 Table Gene Ontology (GO) enrichment analysis of differentially expressed proteins in salivary glands. The p-value was calculated by the software according to the number of proteins included in each GO term in the database, the number of all proteins included in the database, the number of proteins uploaded in this analysis, and the number of proteins enriched in GO term.
(XLSX)

S6 Table Gene Ontology (GO) enrichment analysis of differentially expressed proteins in midguts. The p-value is calculated by the software according to the number of proteins included in each GO term in the database, the number of all proteins included in the database, the number of proteins uploaded in this analysis, and the number of proteins enriched in GO term.
(XLSX)

S7 Table Gene Ontology (GO) enrichment analysis of differentially expressed proteins in ovaries. The p-value is calculated by the software according to the number of proteins included in each GO term in the database, the number of all proteins included in the database, the number of proteins uploaded in this analysis, and the number of proteins enriched in GO term.
(XLSX)

S8 Table Gene Ontology (GO) enrichment analysis of differentially expressed proteins in Malpighian tubules. The p-value is calculated by the software according to the number of proteins included in each GO term in the database, the number of all proteins included in the database, the number of proteins uploaded in this analysis, and the number of proteins enriched inGO term.
(XLSX)

S9 Table KEGG pathway analysis of differentially expressed proteins in salivary glands. GeneRatio: the number of uploaded proteins enriched in the pathway / the number of uploaded proteins; BgRatio: the number of proteins in this pathway in the database / all the numbers in the database; p-value: calculated by constructing a 2 × 2 contingency table based on the above two data; p adjust and q-value are the corrected p-values.
(XLSX)

S10 Table KEGG pathway analysis of differentially expressed proteins in midguts. GeneRatio: the number of uploaded proteins enriched in the pathway / the number of uploaded proteins; BgRatio: the number of proteins in this pathway in the database / all the numbers in the database; p-value: calculated by constructing a 2 × 2 contingency table based on the above two data; p adjust and q-value are the corrected p-values.
(XLSX)

S11 Table KEGG pathway analysis of differentially expressed proteins in ovaries. GeneRatio: the number of uploaded proteins enriched in the pathway / the number of uploaded proteins; BgRatio: the number of proteins in this pathway in the database / all the numbers in the database; p-value: calculated by constructing a 2 × 2 contingency table based on the above two data; p adjust and q-value are the corrected p-values.
(XLSX)

S12 Table KEGG pathway analysis of differentially expressed proteins in Malpighian tubules. GeneRatio: the number of uploaded proteins enriched in the pathway / the number of uploaded proteins; BgRatio: the number of proteins in this pathway in the database / all the numbers in the database; p-value: calculated by constructing a 2 × 2 contingency table based on the above two data; p adjust and q-value are the corrected p-values.
(XLSX)
