# Supplementary figures and images for: Proteomic changes in various organs of Haemaphysalis longicornis under long-term starvation
Source: PLoS Negl Trop Dis. 2022 Aug 22;16(8):e0010692. doi: 10.1371/journal.pntd.0010692 (PMC9394840; doi:10.1371/journal.pntd.0010692)

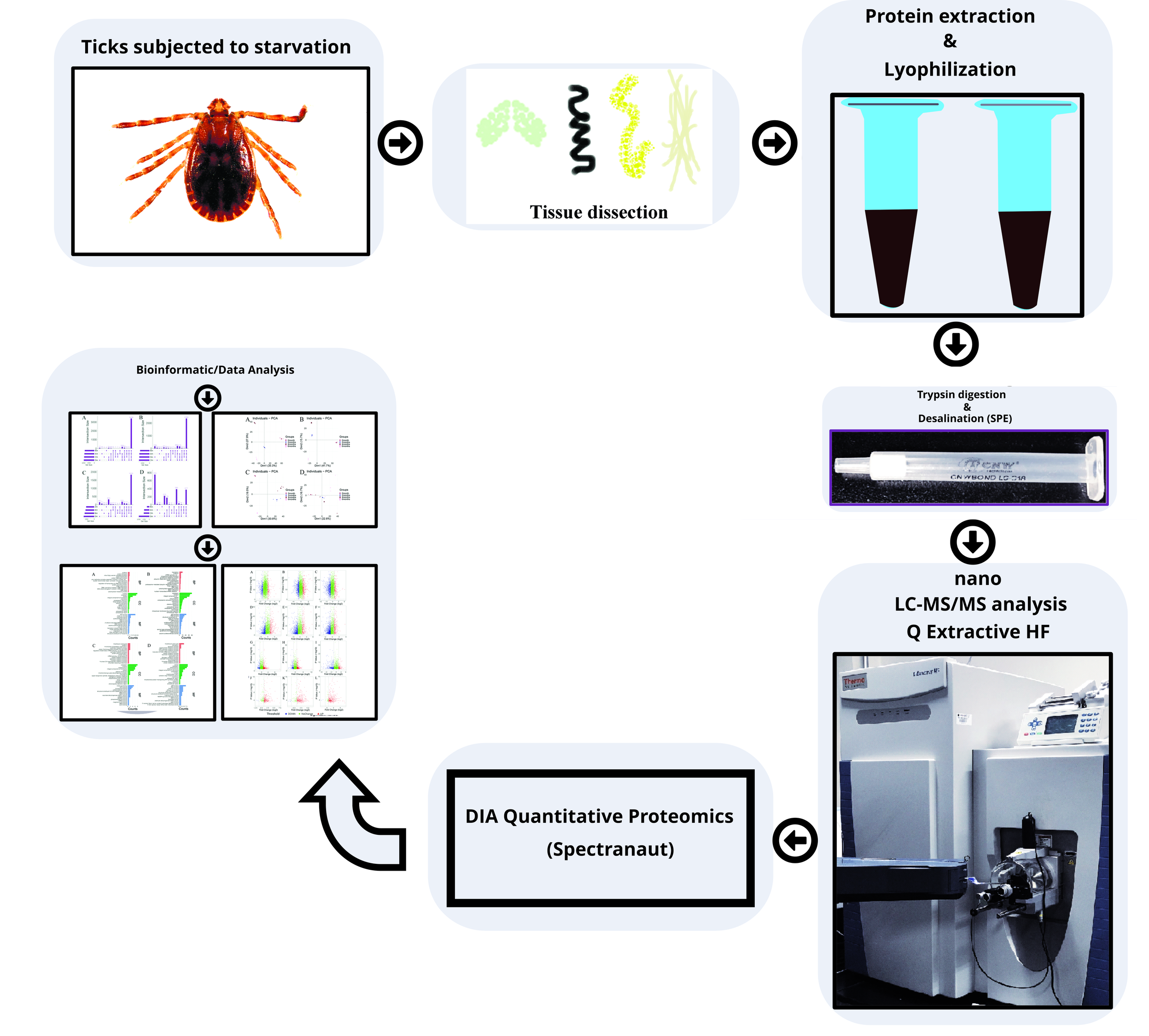

Supplement: S1 Fig — (TIF) [file pntd.0010692.s001.tif]
